# Commentary: “PCRRT Expert Committee ICONIC Position Paper on Prescribing Kidney Replacement Therapy in Critically Sick Children With Acute Liver Failure”

**DOI:** 10.3389/fped.2022.897308

**Published:** 2022-05-02

**Authors:** Akash Deep, Emma C. Alexander, Zaccaria Ricci, Serge Grazioli, Claudio Ronco, Stuart Goldstein, Ayse Akcan-Arikan

**Affiliations:** ^1^Paediatric Intensive Care Unit, King's College Hospital NHS Foundation Trust, London, United Kingdom; ^2^Department of Women and Children's Health, School of Life Course Sciences, King's College London, London, United Kingdom; ^3^Department of Paediatrics, Homerton University Hospital, London, United Kingdom; ^4^Pediatric Intensive Care Unit, Department of Anesthesiology and Critical Care Medicine, Meyer Children's Hospital, Florence, Italy; ^5^Division of Neonatal and Pediatric Intensive Care, Department of Pediatrics, University Hospital of Geneva, Geneva, Switzerland; ^6^Department of Nephrology Dialysis and Transplantation, San Bortolo Hospital, International Renal Research Institute Vicenza (IRRIV), Vicenza, Italy; ^7^Center for Acute Care Nephrology, Cincinnati Children's Hospital Medical Center, Cincinnati, OH, United States; ^8^Divisions of Nephrology and Critical Care Medicine, Department of Pediatrics, Baylor College of Medicine, Houston, TX, United States; ^9^Extracorporeal Liver Support, Texas Children's Hospital, Houston, TX, United States

**Keywords:** acute liver failure, acute-on-chronic liver failure, acute decompensation of cirrhosis, acute kidney injury, pediatric

## Introduction

Acute kidney injury (AKI) in critically sick children is directly associated with increased morbidity and mortality (1, 2). However, the precise incidence of AKI in the setting of liver failure is not well defined. There is an urgent need to describe the epidemiology, risk factors and management of AKI in this cohort of patients.

We therefore read with interest the recent position paper from Raina et al. entitled “*PCRRT Expert Committee ICONIC Position Paper on Prescribing Kidney Replacement Therapy in Critically Sick Children with Acute Liver Failure*”(3). This manuscript presents consensus statements on the management of AKI in pediatric acute liver failure (PALF) by the Pediatric Continuous Renal Replacement Therapy (PCRRT) workgroup and International Collaboration of Nephrologists & Intensivists for Critical Care in Children (ICONIC). These consensus statements are described as being based on a systematic review and meta-analysis of studies of AKI in PALF, as well as Delphi surveys. Based on two meta-analyses of five and three studies respectively, the incidence and mortality of AKI in PALF are estimated. Conservative management options of AKI in PALF are stated to include use of albumin, vasoconstrictors, vaptans. Details of prescription for kidney replacement therapy for AKI in PALF are described including indications, modalities and anticoagulation. In addition, the manuscript describes the current evidence regarding extracorporeal liver support (including MARS, Prometheus, single-pass albumin dialysis, and therapeutic plasma exchange), in PALF.

Since the authors stated that they performed a systematic review of the literature, and that many readers may not have access to the primary articles reviewed, it is extremely important that the studies selected are appropriate, the descriptions are accurate, and the resultant interpretation is supported by the evidence. Given these requirements, we are concerned about the failure to uphold the methodological requirements of a systematic review and meta-analysis as well as conflation of distinct pathologies and are compelled to write this letter to highlight the most concerning aspects of this work.

First and foremost, the authors have used data indiscriminately from studies conducted on patients with either chronic decompensated liver disease (CLD), and acute-on-chronic liver failure (ACLF), to provide recommendations regarding the management of children who develop AKI in PALF. The former two diagnoses are only made in patients with pre-existing chronic liver disease, which renders many of the management recommendations inappropriate, and potentially dangerous. Pediatric liver failure is not a homogenous disease. The clinical pictures of PALF, CLD, and ACLF are virtually discrete and require separate dedicated clinical management approaches with little overlap (4). As per the PALF Study Group definition, cited by the authors, a prerequisite for diagnosis of PALF is absence of known pre-existing chronic liver disease (5). Extrapolation from one disease process to another could have serious, even deadly consequences, as, for example, some medications indicated in the use of ACLF in adults are contraindicated in PALF. Furthermore, evidence generated in adults with cirrhosis is cited. Adult end stage liver disease has an entirely different case mix, predominantly arising from alcohol use disorder in resource rich countries and infectious etiologies in resource limited settings, precluding extrapolation of existing evidence from adult studies to the pediatric realm (6).

Our detailed specific concerns are as follows:

(1) Inaccurate reporting of methodology, and incorrect referencing throughout the paper:

This is most represented in Tables 2, 3. Table 2 is described as showing a meta-analysis of the incidence of AKI in pediatric PALF. Table 2 does not correctly cite the studies used in the meta-analysis (for example, two of the citations displayed in Table 2 link to review articles). Supplement 9 seems to list the correct citations used in the meta-analysis, which includes a study conducted in pediatric liver transplant recipients, two studies including patients with ACLF, and one study in adults with cirrhosis. Without appropriate citations and given that only one of the five studies was conducted in the intended patient population, the meta-analysis gives a misleading impression regarding the incidence of AKI in PALF. Table 3 is described as showing mortality of patients with AKI in children with PALF. Once again, the citations in Table 3 refer to the wrong studies (similar incorrect citations are found elsewhere in the manuscript, such as the section “Citrate”). The correct citations in Supplement 11 reveal the patient populations for this meta-analysis include one study in pediatric liver transplant recipients, one in ACLF, and one in adult patients—therefore *non*e of the included studies targets the correct patient population.

(2) Conflation of different disease processes:

The authors confuse PALF with CLD and ACLF, disease processes that only occur in children with a background of pre-existing liver disease, as well using evidence from patients with inborn errors of metabolism that lead to hyperammonemia in the absence of liver failure.

- Most seriously, the meta-analysis uses studies conducted in ACLF, CLD, and indeed in adults to make recommendations for PALF.
- The authors refer to “hepatic renal syndrome” in PALF. The correct term for functional kidney dysfunction is advanced liver disease is “Hepatorenal Syndrome” and is defined as AKI by KDIGO creatinine criteria with the prerequisite presence of cirrhosis and ascites. This is therefore distinct from AKI in PALF and has separate and unique management considerations due to different pathophysiology.
- The recommendation of using high volume hemofiltration cite studies from Spinale et al. (7) and Hanudel et al. (8), both on neonatal hyperammonaemia which results from a congenital metabolic defect that leads to extremely elevated serum ammonia concentrations, and eventually to severe neurological defects if not reduced expeditiously. Therefore high effluent rates, either as continuous veno-venous haemodialysis (CVVH) or continuous veno-venous haemodiafiltration (CVVHD) are recommended and this intervention is considered as an emergency. High dose emergent extracorporeal clearance used in inborn errors of metabolism to limit neurotoxicity related to severe elevations of serum ammonia are not directly translatable to AKI management of PALF.

(3) Inappropriate recommendations regarding the pharmacologic management of PALF:

- In the section on conservative management of AKI in PALF, the authors mention interventions for these patients includes “*albumin, vasoconstrictors and vaptans*”. This recommendation has the potential to cause harm if used in patients with AKI in PALF. Because of the unique pathophysiology of HRS in chronic liver disease, these are drugs specifically designed to be used in HRS characterized by fluid overload and hyponatremia resistant to loop diuretics, and not in AKI of PALF. If given to patients with AKI of PALF, refractory polyuria could ensue with severe dehydration and electrolyte complications. Vaptans could be severely hepatotoxic and cannot be recommended outside the confines of a clinical trial with rigorous adverse event reporting structure and safety oversight in PALF.
- The referenced study from Saxena et al. (“Use of terlipressin in critically ill children with liver disease” (9), was conducted in children with CLD where the response of terlipressin on serum creatinine in children with HRS-AKI or non-HRS-AKI was compared. None of the patients in this study had PALF. Terlipressin is not used in AKI of PALF because splanchnic vasodilatation, which is a hallmark of HRS of CLD due to portal hypertension, is not present in PALF.
- In a similar context, albumin works as an antioxidant, volume expander and immune modulator specifically in patients with cirrhosis and HRS (10). It does not have a role in staging in AKI in PALF.
- The recommend hydrocortisone dose in AKI of PALF of “*1–2 mg/kg/dose of hydrocortisone administered six times per hour”* is incorrect—hydrocortisone should be administered once every 6 h and not six times per hour. Further, hydrocortisone is not recommended for patients with AKI, but for patients with ALF in multi-organ failure with relative adrenal insufficiency (11).

## Conclusions

The recommendations put forth in this manuscript are not based on an accurate representation of the patient population for whom the authors target and could have grave consequences of causing harm in the care of critically ill children with PALF who have AKI.

We have listed the general principles for managing AKI in PALF, according to the expert opinion of these co-authors, in Table 1.

**Table 1:** General principles in managing acute kidney injury in paediatric acute liver failure.

• AKI can occur in patients with PALF, CLD or ACLF. • It is very important to distinguish between these three entities as the treatment modalities are considerably different owing to the varying pathophysiology of each liver condition. • The first question for a clinician is to decide whether the liver failure is acute, chronic, or acute-on-chronic. • The aetiology of AKI in PALF is multifactorial - hypovolaemia due to various causes [decreased fluid intake, increased losses (diarrhoea, vomiting)], hypoperfusion related acute tubular injury, toxins affecting both the liver and kidney simultaneously, inflammatory insult from damage-associated molecular patterns released from the failing liver or pathogen-associated molecular patterns translocating from the gut or intra-abdominal hypertension (12). • Diagnostic criteria for AKI in PALF are identical to AKI in patients with non-liver diseases (13). • The presence of AKI in children with PALF further impairs ammonia clearance and potentially augments the adverse effects of ammonia on the brain. • Conservative management of AKI in PALF is the same as for AKI in any non-liver disease - management of euvolemia, preventing fluid overload, maintaining renal perfusion, cautious use of nephrotoxic drugs. • In addition to the standard indications for continuous KRT (CKRT) in patients with AKI in PALF i.e., fluid overload, oliguria, refractory electrolyte and acid base abnormalities (14), CKRT is also initiated in PALF for non-renal indications, especially hyper-ammonaemia and hepatic encephalopathy Grade 3/4. • Patients with AKI in PALF can have increased morbidity and mortality (15) including a potential effect on spontaneous regeneration of the liver and adverse effects post-transplantation.

## Author Contributions

AD, SG, AA-A, and ZR conceived of the article, wrote the first draft, and led subsequent revisions. All authors contributed to the final version of the manuscript submitted for publication.

## Conflict of Interest

The authors declare that the research was conducted in the absence of any commercial or financial relationships that could be construed as a potential conflict of interest.

## Publisher's Note

All claims expressed in this article are solely those of the authors and do not necessarily represent those of their affiliated organizations, or those of the publisher, the editors and the reviewers. Any product that may be evaluated in this article, or claim that may be made by its manufacturer, is not guaranteed or endorsed by the publisher.

## Abbreviations

ACLF: Acute-on-Chronic Liver Failure
AKI: Acute Kidney Injury
CKRT: Continuous Kidney Replacement Therapy
CLD: Chronic Decompensated Liver Disease
CVVH: Continuous Veno-Venous Haemodialysis
CVVHD: Continuous Veno-Venous Haemodiafiltration
HRS: Hepatorenal Syndrome
KDIGO: Kidney Disease: Improving Global Outcomes
PALF: Pediatric Acute Liver Failure.
